# Theoretical Analysis of Terahertz Dielectric–Loaded Graphene Waveguide

**DOI:** 10.3390/nano11010210

**Published:** 2021-01-15

**Authors:** Da Teng, Kai Wang

**Affiliations:** 1 College of Physics and Electronic Engineering, Zhengzhou Normal University, Zhengzhou 450044, China; 2 Key Laboratory of Infrared Imaging Materials and Detectors, Shanghai Institute of Technical Physics, Chinese Academy of Sciences, Shanghai 200083, China; wangkai@mail.sitp.ac.cn

**Keywords:** graphene plasmons, waveguides, subwavelength structures, terahertz waves

## Abstract

The waveguiding of terahertz surface plasmons by a GaAs strip-loaded graphene waveguide is investigated based on the effective-index method and the finite element method. Modal properties of the effective mode index, modal loss, and cut-off characteristics of higher order modes are investigated. By modulating the Fermi level, the modal properties of the fundamental mode could be adjusted. The accuracy of the effective-index method is verified by a comparison between the analytical results and numerical simulations. Besides the modal properties, the crosstalk between the adjacent waveguides, which determines the device integration density, is studied. The findings show that the effective-index method is highly valid for analyzing dielectric-loaded graphene plasmon waveguides in the terahertz region and may have potential applications in subwavelength tunable integrated photonic devices.

## 1. Introduction

The terahertz (THz) wave, usually defined as a frequency ranging from 1 to 10 THz, has attracted numerous research interests due to its potential applications in the fields of spectroscopy, imaging, defense industries, on-chip communications [1,2,3,4,5,6], etc. In particular, guiding the THz wave is a challenge for applications in communications; thus, the THz waveguide has become a hot research topic. By using THz waveguides, THz waves can be easily concentrated in the subwavelength region, offering a tightly confined modal field beyond the diffraction limit [7]. Earlier, dielectric waveguides [8,9,10,11] and noble metal-based structures [12,13,14,15,16,17,18,19,20,21,22,23,24,25] were widely investigated for THz waveguiding, such as hollow-core [10] and porous-core dielectric waveguides [11], metallic nanowires [13,14,15,16,17,18,19,20,21,22,23,24,25], metal-based hybrid waveguides [26,27,28], etc. THz dielectric waveguides could realize a low loss mode propagation; however, the modal fields are diffraction-limited. In contrast to dielectric waveguides, metallic waveguides could support the transverse magnetic (TM) surface plasmon (SP) mode [29], while in the THz range, the SP effects of metal are relatively weak [30], thus hindering the applications at a subwavelength scale.

Recently, it was reported that graphene could also excite SP modes [31,32,33] in the infrared band, offering an alternative approach to guiding THz waves. Compared with the metallic waveguides, graphene plasmon waveguides could confine the infrared waves into the deep subwavelength scale, and the guided modes could be easily tuned [34]. Up to now, lots of graphene-based configurations have been proposed to guide the infrared waves, such as graphene ribbons [35], graphene slot waveguides [36,37], graphene wedge and groove waveguides [38], dielectric-loaded graphene waveguides [39], graphene-coated nanowires [40,41,42,43,44,45,46], graphene-based hybrid waveguides [47,48], etc.

In the THz band, Huang et al. [49] proposed a graphene-coated nanowire with a drop-shaped cross section to realize low loss waveguiding with an ultra-strong mode confinement. Additionally, a graphene-coated elliptical nanowire was suggested for ultra-deep subwavelength THz waveguiding [50]. Zhou et al. [51] proposed a graphene-based hybrid plasmonic waveguide to achieve ultra-deep subwavelength modal field confinement. A symmetric hybrid plasmonic waveguide [52] was proposed to achieve a propagation length of 26.7 mm and mode area of about 4 μm^2^ at 10 THz. Later, Wan et al. [53] proposed a dielectric-loaded graphene groove waveguide, and a typical propagation length of about 37.8 μm and mode area of about 52 μm^2^ were obtained at 1.5 THz. To achieve a better modal field confinement, two graphene-based hybrid plasmonic waveguides were proposed [54,55], which could simultaneously achieve an ultra-small modal area and a propagation length of about several hundreds of micrometers at 3 THz. Despite recent progress in graphene-based waveguides, we note that the current reports mainly focus on the near- and mid-infrared band, and graphene-based THz waveguides are studied less, relatively speaking. Furthermore, to the best of our knowledge, the modal properties and crosstalk in dielectric-loaded graphene surface plasmon waveguides (DLGSPWG) have not yet been fully investigated in the THz region.

In this paper, we extend the concept of the dielectric-loaded plasmon waveguide [39,56,57,58], which dates back to 2006, from the near- and mid-infrared band to the THz band by proposing a THz DLGSPWG. Although the physics behind this are similar to those of [39,58], our findings show something new. The DLGSPWG could be easily fabricated, and the substrate structures can provide additional freedom to tune the modal properties. Based on the effective-index method (EIM) [58,59,60], we first give an approximate analytical model for the DLGSPWG and then calculate the effective mode index, propagation length, and the cut-off wavelength of higher order modes. The group velocity of the propagated modes and crosstalk between adjacent structures are also discussed. Our findings show that the EIM is highly valid for analyzing DLGSPWG in the THz region, and the results are verified by the numerical simulations based on the finite element method (FEM). The results also show that the electromagnetic field in the corner regions is only partly responsible for the difference between the EIM and FEM results and that the crosstalk between adjacent structures is negligible even at a very small separation distance.

## 2. Theory

**Figure 1:**
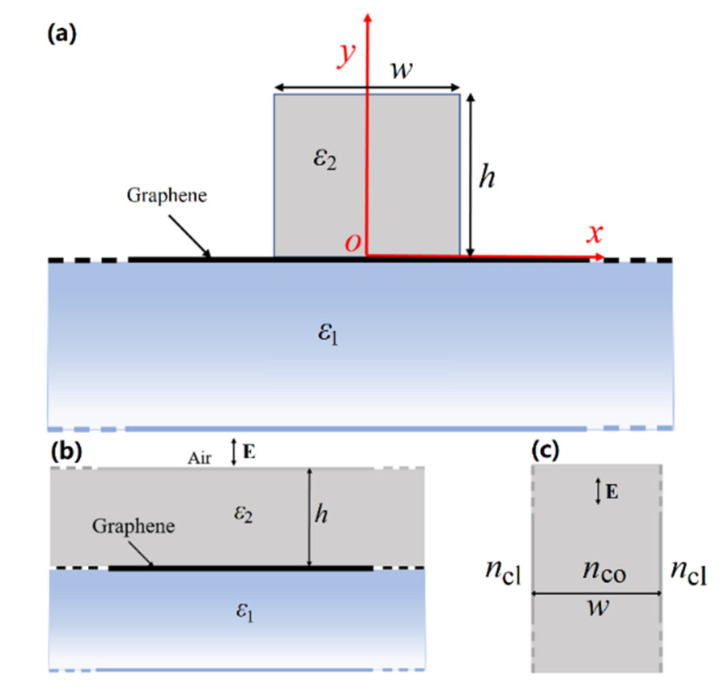
(**a**) Cross-section of the DLGSPWG structure. (**b**) The equivalent four-layer and (**c**) three-layer structures considered in the EIM.

As seen from Figure 1a, the proposed DLGSPWG consists of a GaAs dielectric strip with a width of *w* and height of *h* located on the graphene layer above a semi-infinite dielectric substrate. Figure 1b,c show the equivalent four- and three-layer a structures considered in the EIM. The relative permittivities of the GaAs strip (*ε*_2_) and substrate (*ε*_1_) are set to be 12.25 [54,55]. The range of frequency is from 2 to 10 THz.

We first employ EIM to give an approximate analytical model for the DLGSPWG and then verify the results by using the FEM simulation. In the EIM, the 2D cross-section of the DLGSPWG is divided into two 1D waveguide structures (Figure 1b,c). By individually solving the guided modes of these two 1D waveguides [58], one can finally obtain the guided modes of the DLGSPWG. The proposed DLGSPWG (Figure 1a) could be obtained by narrowing the width of the top GaAs dielectric layer (Figure 1b). Then, we can calculate the effective mode index *n*_co_ of the guided modes in such an equivalent four-layer structure. All the dimensions are infinite along the *x* axis, and the upper air and substrate dielectric layers are semi-infinite along the *y* direction. Here, *n*_co_ is irrelevant to the width of the dielectric strip and is equivalent to that of the effective mode index of the SP mode supported by the air-dielectric-graphene-dielectric structure (Figure 1b), which is given as [31] $n_{\mathrm{co}}=\varepsilon_{0}(\varepsilon_{1}+\varepsilon_{2})ci/\sigma_{g}$, where *ε*_0_ is the permittivity in air, *σ*_g_ is the optical conductivity of graphene, and *c* is the speed of light. Then, the GaAs strip serves as the core of a three-layer dielectric waveguide shown in Figure 1c. The effective mode index *n*_cl_ is equivalent to that of the SP mode supported by the graphene-air interface and is given by [31] $n_{\mathrm{cl}}=\varepsilon_{0}(\varepsilon_{1}+1)ci/\sigma_{g}$. Finally, the eigen-equation of the equivalent dielectric planar waveguide for the *N*-th order guided transverse electric (TE) mode (i.e., TM plasmon mode) is given as [56]:

(1) $$\tan(\frac{k_{1}w-N\pi }{2})=k_{2}/k_{1},N=0,1,2\ldots$$

where $k_{1}=k_{0}\sqrt{n_{\mathrm{co}}^{2}-n_{\mathrm{neff}}^{2}}$, $k_{2}=k_{0}\sqrt{n_{\mathrm{neff}}^{2}-n_{\mathrm{cl}}^{2}}$, *k*_0_ = 2π/*λ*_0_, *λ*_0_ is the wavelength of incident light in free space. *w* is the width of the dielectric strip, and *n*_eff_ is the effective mode index of the propagating modes in DLGSPWG. By numerically solving Equation (1), the effective mode index *n*_eff_ of the *N*-th order mode could be obtained. The real part of *n*_eff_ is related to the dispersion, and the imaginary part Im(*n*_eff_) is related to the modal loss. The power propagation length *L*_P_ is defined as *L*_P_ = 1/2α, where α is the loss factor related to Im(*n*_eff_) and given by α = *k*_0_Im(*n*_eff_). Therefore, the power propagation length could be calculated by *L*_P_ = *λ*_0_/[4πIm(*n*_eff_)]. The cut-off condition of the guided modes is *k*_2_ = 0. Then, the cut-off wavelength of the *N*-th order guided mode can be calculated by [58,60]:

(2) N⋅λcutoffN=2w⋅Re(nco2−ncl2), N=1,2,3…

Here, the impact of the strip height *h* (assumed to be infinite) has not been taken into account in the EIM, since graphene plasmons are tightly concentrated at the interface of the graphene layer. In the FEM calculation, the height of the dielectric strip is *h* = 2 μm, which is enough to guarantee the accuracy. Furthermore, enlarging *h* will be much closer to the situation in EIM and will lead to a better match between the EIM and FEM results, especially for the fundamental mode. We will show this subsequently.

In the calculation, the graphene layer is modelled as an electric field-induced surface current **J** = *σ*_g_**E** on the surface of the substrate, thus neglecting the thickness of the graphene layer. Within the random-phase approximation, the complex optical conductivity of graphene [61,62,63] consists of the interband and intraband contributions in the THz range, that is *σ*_g_ = *σ*_intra_ + *σ*_inter_, with:

(3) $$\sigma_{\mathrm{intra}}=\frac{2ie^{2}k_{B}T}{\pi \hbar^{2}(\omega +i/\tau )}\ln[2\cosh(\frac{E_{F}}{2k_{B}T})]$$

(4) $$\sigma_{\mathrm{inter}}=\frac{e^{2}}{4\hbar^{}}[\frac{1}{2}+\frac{1}{\pi }\arctan(\frac{\hbar \omega -2E_{F}}{2k_{B}T})-\frac{i}{2\pi }\ln\frac{{\left(\hbar \omega +2E_{F}\right)}^{2}}{{\left(\hbar \omega -2E_{F}\right)}^{2}+{\left(2k_{B}T\right)}^{2}}]$$

Here, we set *τ =* 0.5 ps (unless otherwise mentioned) for the electron relaxation time and *T =* 300 K for the temperature. *E*_F_ is the Fermi energy level of the graphene, *ω* is the angular frequency of incident light, *ħ* is the reduced plank constant, *k_B_* is the Boltzmann’s constant, and *e* is the charge of the electron. 

The FEM results are obtained by use of the wave optics module of COMSOL Multiphysics. The eigenvalue solver is used to find modes of the waveguide. The calculation domain is 2*λ*_0_ × 2*λ*_0_, and a perfectly matched layer (PML) is applied around the geometry to avoid the influence of reflection. A convergence analysis is also conducted to ensure that the numerical boundaries and meshing do not interfere with the solutions.

**Figure 2:**
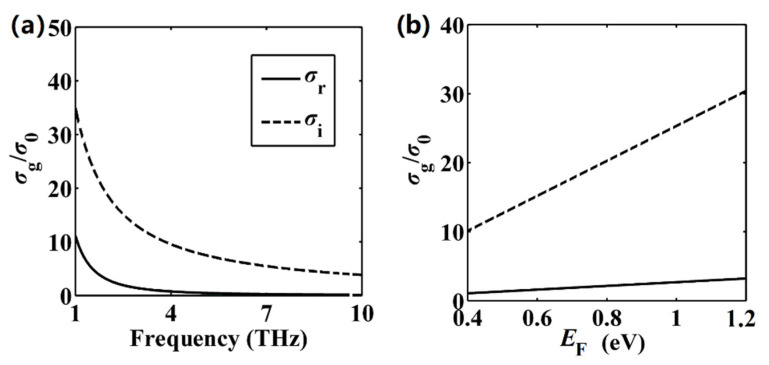
Graphene conductivity *σ*_g_ normalized by *σ*_0_ = *e*^2^/*ħ* based on Equations (3) and (4). Real and imaginary parts of *σ*_g_ as functions of the (**a**) frequency and (**b**) Fermi energy level. *E*_F_ = 0.5 eV for (**a**), *f* = 3 THz for (**b**).

In the derivation of Equation (1), we assume that graphene can support and propagate TM plasmon modes. Actually, graphene can support TM plasmon modes when Im(*σ*_g_) > 0 [32]. To illustrate this, we plot *σ*_g_ with respect to the frequency and *E*_F_ in Figure 2. Obviously, within the frequency region (2–10 THz, i.e., corresponding wavelength region 30–150 μm) and Fermi energy level region considered here, the imaginary parts of *σ*_g_ (Im(*σ*_g_) = *σ*_i_) are always above zero, thus indicating that TM plasmon modes could be excited.

## 3. Results and Discussion

**Figure 3:**
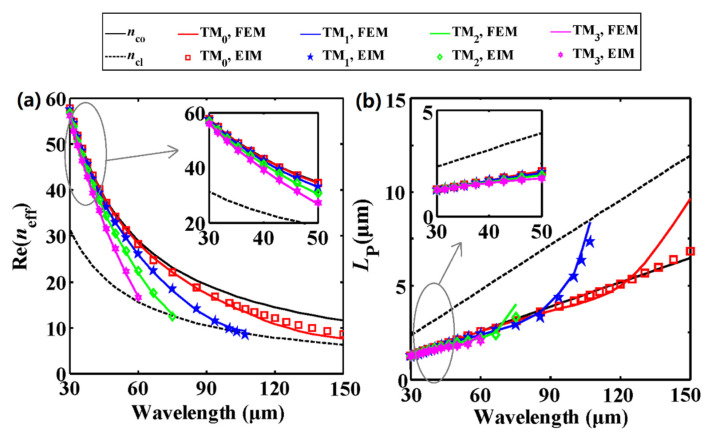
(**a**) Effective mode indices and (**b**) propagation lengths of the SP modes in DLGSPWG. The insets of (**a**) and (**b**) show the details at shorter wavelengths. Solid lines for FEM, and symbols for the EIM calculations. The solid and dashed black lines represent *n*_co_ and *n*_cl_. *w* = 4 μm, *h* = 2 μm, *E*_F_ = 0.6 eV.

By analytically solving Equation (1), we first investigate the effective mode indices of the SP modes supported by the DLGSPWG, and then verify the analytical results by using the FEM simulation. In the calculation, the width and height of the dielectric strip are *w* = 4 μm and *h* = 2 μm. As seen from Figure 3, the EIM results (symbols) are in good agreement with the FEM (solid lines) results when the wavelength is smaller than the cut-off wavelength. The fundamental plasmon mode (*N* = 0, TM_0_) has a larger real part of the effective mode index (Re(*n*_eff_)) than the higher order modes (*N* = 1, 2, 3), which implies a shorter wavelength (*λ*_SP_ = *λ*_0_/Re(*n*_eff_)), as shown in Figure 3a. All the mode indices are located between *n*_co_ and *n*_cl_. When increasing the wavelength from 30 to 150 μm, Re(*n*_eff_) decreases monotonically and the higher order modes are gradually cut off. The power propagation length increases monotonically, which suggests a smaller modal loss at longer wavelengths (Figure 3b). 

**Figure 4:**
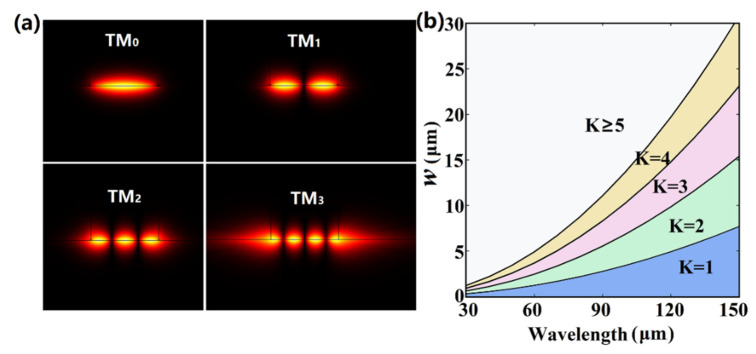
(**a**) Modal fields distributions (|*E*_y_*|*) of the first four order modes at *λ*_0_ = 60 μm (5 THz) with *w* = 4 μm and *h* = 2 μm. (**b**) The fundamental mode and multi-modes operation regions calculated by Equation (2) as functions of the strip width *w* and the wavelength. K represents the total numbers of guided modes. *E*_F_ = 0.6 eV.

As the wavelength is close to the cut-off wavelength, the EIM results show slight deviations from the FEM results. This is due to the fact that the electromagnetic fields in the corner regions have not been taken into account in the EIM. At shorter wavelengths, the modal fields are perfectly localized in the GaAs strip. Therefore, the EIM results are highly consistent with the FEM simulation results. Figure 4a shows the modal fields (|*E*_y_|) of the first four order modes at the wavelength of 60 μm (5 THz), and one can see that the fields are perfectly localized below the GaAs strip. However, at longer wavelengths, the modal fields are not perfectly localized, which leads to slight difference between these two methods.

The fundamental plasmon mode is cut-off-free and has a lower modal loss compared with higher order modes and could thus be used for long-range propagation. Here, the waveguide could also support higher order modes, and we next study the single and multi-mode operation regions by solving Equation (2). As shown in Figure 4b, at a certain wavelength the numbers of guided modes (K) increase with an increasing width of the GaAs strip. The critical values of *w* for the single-mode operation are 0.3073 μm and 7.6937 μm for *λ*_0_ = 30 and 150 μm, respectively. As *w* increases, the single-mode operation region tends towards a longer wavelength. At a certain width, the number K decreases with an increase in the wavelength. Finally, single mode propagation could be achieved at a very small *w* and long wavelength.

As depicted in Figure 3, the EIM results (symbols) are in very good agreement with the FEM (solid lines) results when the wavelength is smaller than 120 μm. Therefore, the wavelength ranges from 30 to 120 μm (i.e., 2.5–10 THz) in what follows.

**Figure 5:**
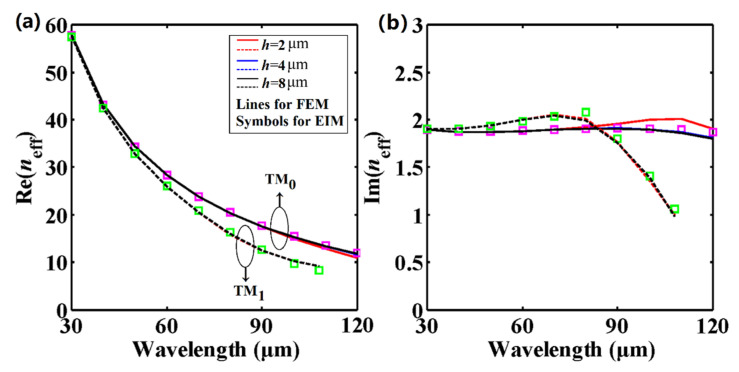
(**a**) Real and (**b**) imaginary parts of the effective mode indices of the TM_0_ and TM_1_ modes in DLGSPWG with respect to the wavelength at different strip heights. *w* = 4 μm, *E*_F_ = 0.6 eV. Note that the blue and black lines nearly overlap each other. Solid lines and pink squares for TM_0_, dashed lines and green squares for TM_1_.

In Section 2, it is mentioned that the impact of the strip height *h* (assumed to be infinite) has not been taken into account in the EIM, while in the FEM calculation the height of the dielectric strip is *h* = 2 μm. To make the structure used in the numerical simulation much closer to that used in EIM, we have studied the effective mode indices with respect to the wavelength at different strip heights *h* for the TM_0_ and TM_1_ modes, as shown in Figure 5. We find that the electromagnetic field in the corner regions is only partly responsible for the difference between the EIM and FEM results. By enlarging *h*, these two results are in better agreement with each other, even at longer wavelengths. As seen from Figure 5, the FEM results with a larger strip height (blue (*h* = 4 μm) and black lines (*h* = 8 μm)) have smaller deviations compared with the EIM results (symbols) for the TM_0_ mode. For instance, when *h* = 2 μm and the wavelength is 120 μm, the relative deviation of Re(*n*_eff_) between FEM and EIM is about 9.9%, while the relative deviation is only 2.7% for *h* = 8 μm. As for the imaginary parts of *n*_eff_, the relative deviation is always less than 6%. Interestingly, the strip height seems to have little effect on the effective mode indices for the TM_1_ mode, and the EIM results are always in good agreement with the FEM results, even at different *h* values. 

**Figure 6:**
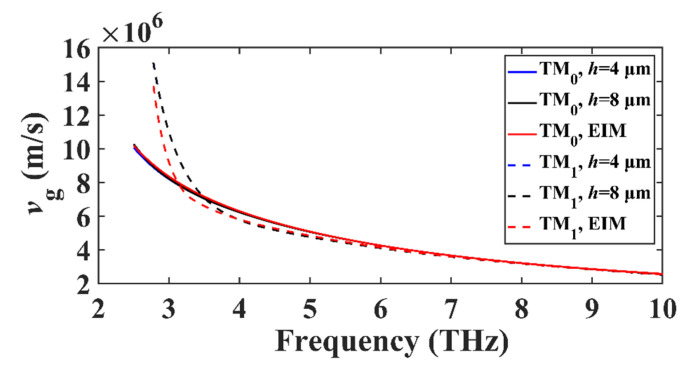
Group velocity *v*_g_ of the TM_0_ and TM_1_ modes in DLGSPWG with respect to the frequency. *w* = 4 μm, *E*_F_ = 0.6 eV. Note that the blue and black lines nearly overlap each other. Solid lines for TM_0_ and dashed lines for TM_1_.

Next, we study the group velocity, defined as $v_{g}=\partial \omega /\partial (k_{0}n_{\mathrm{eff}})$, of the propagated THz plasmon modes. As shown in Figure 6, the group velocity of the TM_0_ mode gradually decreases with an increasing frequency, which is due to the fact that Re(neff)+ω∂Re(neff)/∂ω (that is, $c/v_{g}$) is much larger at a higher frequency. As for the TM_1_ mode, the group velocity first rapidly decreases and then approaches that of TM_0_. One can see that the EIM results are consistent with the FEM results.

**Figure 7:**
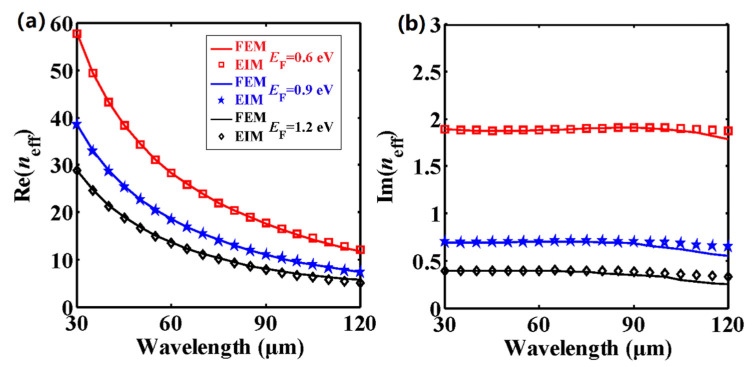
(**a**) Real and (**b**) imaginary parts of the effective mode indices of the fundamental mode in DLGSPWG with respect to the wavelength at different *E*_F_ values. *w* = 4 μm, *h* = 8 μm.

One of the outstanding properties of graphene is the tunability of the surface conductivity by employing a DC bias [64], and this therefore provides us with a feasible method for modulating the effective mode indices of the guided modes. Figure 7 shows the effective mode indices of the fundamental mode in DLGSPWG with respect to the wavelength at different *E*_F_ values. As we have mentioned above, one could get a better fitting between the FEM and EIM results by enlarging the strip height at longer wavelengths. Hence, *h* is set to be 8 μm. The carrier density in graphene could reach a value as high as 10^14^ cm^−2^ [65], and the corresponding *E*_F_ is about 1.17 eV. In a relatively recent work [66], the Fermi energy level reached a value as high as 1.77 eV. Here, *E*_F_ varies from 0.6 eV to 1.2 eV. It is worth mentioning that we set *τ =* 0.5 ps in the above investigations. Actually, *τ* is related to *E*_F_ and is given by *τ = nE*_F_/*eV*_F_^2^ [67,68], where *n* = 10,000 cm^2^/(V·s) is the carrier mobility of graphene and *V*_F_ = 10^6^ m·s^−1^. As a result, *τ* is 0.9 (1.2) ps for *E*_F_ = 0.9 (1.2) eV. From Figure 7a, one can see that at a fixed wavelength, Re(*n*_eff_) decreases as *E*_F_ increases. For *E*_F_ = 0.9 eV, the analytical results of Re(*n*_eff_) range from 38.49 to 7.24. Clearly, the EIM results are in good agreement with the FEM results. In terms of the imaginary parts, shown in Figure 7b, when enlarging *E*_F_, Im(*n*_eff_) decreases dramatically, which is due to the fact that the interband contribution of *σ*_g_ is reduced by increasing the Fermi energy level. Thus, the propagation length could be massively enhanced. However, the EIM results show a slight discrepancy with the FEM results at longer wavelengths. This is because the electromagnetic fields in the corner regions have not been taken into account in the EIM; thus, the modal loss is slightly larger than for the FEM results.

**Figure 8:**
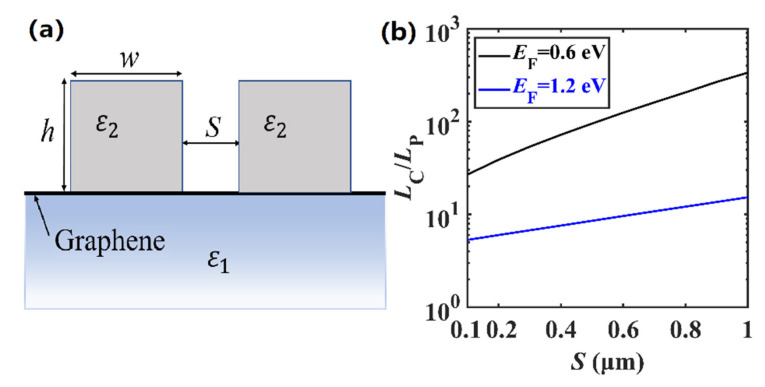
(**a**) Coupling system with two DLGSPWG structures; and (**b**) Normalized coupling length *L*_C_/*L*_P_ with respect to *S* at different *E*_F_ values. *w* = 4 μm, *h* = 4 μm, and *f*_0_ = 5 THz.

Besides the modal properties, crosstalk between the adjacent waveguides, which determines the device integration density, is also an important factor in subwavelength photonic integration. In order to investigate the crosstalk, we set a coupling system with two DLGSPWG structures separated by a distance of *S*, as shown in Figure 8a. To evaluate the performance of the coupling system in the integrated THz circuit, crosstalk is characterized by using a coupling length *L*_C_ [69], which is calculated by *L*_C_ = π/(|*β*_s_ − *β*_as_|), based on the coupled mode theory [69], where *β*_s_ = *k*_0_*n*_eff,s_ and *β*_as_ = *k*_0_*n*_eff,as_ are the complex propagation constants of the symmetric and antisymmetric modes at 5 THz, respectively. Figure 8b shows the normalized coupling length *L*_C_/*L*_P_ with respect to *S*. The dependence of the coupling length *L*_C_ on *S* indicates that the crosstalk would be weaker with the increase of *S*. To ensure a very low crosstalk, *L*_C_/*L*_P_ needs to be large enough (usually exceeding 10). This means that the energy of the mode decays to 1/*e* of its original value before it is coupled to the adjacent structure; thus, the crosstalk between adjacent structures is negligible. Here, even at a very small separation distance of *S* = 0.1 μm, *L*_C_/*L*_P_ is as high as 26.89 for *E*_F_ = 0.6 eV; thus, the crosstalk can almost be ignored. However, for *E*_F_ = 1.2 eV, *L*_C_/*L*_P_ is about 10 for *S* = 0.6 μm. The low-mode crosstalk between the adjacent waveguides shows its potential application in ultra-compact and high-density integration.

## 4. Conclusions

We extend the concept of dielectric-loaded plasmon waveguide from the near- and mid-infrared band to the THz band. The SP modes of the DLGSPWG are studied by using the EIM and FEM. The tunability of the fundamental mode, effective mode index, propagation length, and the cut-off wavelength of higher order modes are investigated. The EIM results are in good agreement with the FEM results in the THz band. Our findings show that the electromagnetic field in the corner regions is only partly responsible for the difference between the EIM and FEM results. Enlarging the strip height will lead to a better fitting between the EIM and FEM results, especially for the fundamental mode. Besides this, we show that the DLGSPWG shows very small crosstalk between the adjacent structures, even at a very small separation distance of 0.1 μm. The DLGSPWG may have potential applications in tunable subwavelength terahertz photonic devices.

## Data Availability

The data presented in this study are available on request from the corresponding author.
